# An Account of the Autumnal Fever of Georgia, from Observations Made during Four Years, in the Counties of Jones and Baldwin

**Published:** 1829

**Authors:** James C. Finley

**Affiliations:** Cincinnati


WESTERN JOURNAL

OF THE

### MEDICAL & PHYSICAL SCIENCES.

JULY, AUGUST, & SEPTEMBER, 1829.

### ESSAYS AND CASES.

ART. I.—*An account of the Autumnal Fever of Georgia, from observations made during four years, in the counties of Jones and Baldwin.* By JAMES C. FINLEY, M. D. now of Cincinnati.

THOSE forms of autumnal fever which ravage the Southern states exhibit very different symptoms in different sections of the country. On the seaboard, they are attended with great determinations to the stomach, and that peculiar train of symptoms, (occasionally assuming the malignant character,) which accompany affections of this organ. On the borders of large rivers which overflow their banks, and in the neighborhood of extensive marshes, they are accompanied by long and severe chills, with great enlargement of the liver and spleen. In the middle region, or that section of country included between the low country and the mountainous region, they exhibit a modified character, and are generally attended with febrile symptoms of a more open and inflammatory nature. But notwithstanding this general ground of distinction, local and accidental circumstances may produce every variety of the disease in any situation.

The epithet Bilious, as applied to this species of fever, if properly restricted, and distinctly understood to apply only to a prominent symptom, and not to the cause of the disease, is perhaps unobjectionable; and as it has been appropriated to that species of fever which proceed from marsh miasmata, it forms a distinctive appellation readily understood.

It is however to be borne in mind, that the liver is not the principal seat of the disease, but is affected only in common with other organs, and often in an inferior degree; and that the secretion of bile, so far from being universally increased, is frequently diminished, and sometimes entirely suspended.

The section of country from which the following remarks have been drawn, is situated between the Oconee and Oakmulgee rivers, about a hundred miles from their junction. The soil is fertile, the face of the country but little broken, and the streams of water, confined within shallow banks and descending by a slow and easy course, are liable to overflow and form marshes of considerable extent. This peculiarity in the features of the country is always to be observed in those situations which are subject to the various forms of autumnal disease. Where the soil is barren, or where the irregularities of the surface afford the streams a rapid descent, the situations may generally be considered healthy. Hence the mountainous region in the northern and western parts of the Southern states are almost universally healthy. The pine woods, which, (with the exception of the margins of water courses,) have a barren soil, are free from the annual visitation of intermittent fever. On the contrary, sections of country, which by their fertility of soil and advantageous situation, are peculiarly favorable to agricultural pursuits, are every year infested with autumnal fever. These facts are so familiar to every one acquainted with the topography and diseases of the Southern states, as to require no further illustration.

*Remote causes of the disease.*—The influence of marshes in producing Intermittent and Remittent fevers is so well known and so generally acknowledged, that it would be superfluous to advance any thing upon this subject. In addition to this, the decay of vegetable matter attendant upon the clearing of new countries is supposed by many to be a fruitful source of autumnal disease. Facts are not wanting which apparently tend to favor this opinion very strongly. Mill ponds when first erected, filled as they usually are with the trunks and branches of trees, generally exert a more deleterious influence upon the health of the surrounding country than they do after a lapse of a few years. New countries, in which extensive clearings have been made, are frequently more unhealthy within the first five or six years after they are cleared than they are at any subsequent period. Green wood and other vegetable matter confined in unventilated situations, as the holds of ships, &c. are frequently a fatal source of malignant fevers. Judge Clayton, of Athens, informed me that at his plantation in Clarke county, on which from its elevated situation a case of autumnal fever was never known to originate, the family of his overseer, a few years ago, were successively seized with a bilious fever of a very obstinate nature, for the origin of which they could account in no other way but by the decayed condition of the logs of which the house was constructed. Other facts of a similar nature have fallen within my own observation; and upon a superficial view, appear to favour the popular opinion of the deleterious effects of vegetable decomposition. A closer investigation of the subject, however, will lead us to the conclusion, that this opinion is unfounded, or, at least, that the elements of vegetable matter in marshy situations acquire a malignity which is not necessarily attached to them.

Many of the facts stated above admit of a different explanation, and many circumstances can be adduced to invalidate the conclusion drawn from the rest.

1st. The operations of the animal economy are enveloped in so much obscurity, that when a number of causes concur in the production of disease, it is difficult to say how much importance is to be attributed to any one of them. In the investigation of the causes of bilious fever we feel this difficulty in its full extent. The origin and nature of miasmata, their mode of operation, and the influence of exciting causes, are so little understood that they open a wide field for conjecture and speculation, and when we have investigated the sources of this disease to the utmost extent, that our limited range of observation admits of, we must still believe that our conclusions are but approximations to the truth. Change of climate and long established habits, unwholesome provisions, the fatigue and exposure attendant on clearing and cultivating new lands, the want of many of the conveniences of life, and the melancholy arising from the want of society, and the loss of friends, must of themselves frequently prove an abundant source of disease among new settlers.

2dly. A country in its natural state covered with forests, abounds with marshes to a much greater extent than when it is cleared and cultivated, and it is reasonable to suppose that the consequences of exposing these marshes to the sun will be very unfavorable to the salubrity of the climate.

3dly. Marshes, when covered with vegetation sufficiently luxuriant to protect them from the influence of the sun, are comparatively innocent. Situations in the neighbourhood of mill ponds, which have suffered severely for a succession of years from fever, become healthy when the pond is drawn off sufficiently early to allow the rank vegetation usually covering such places to spring up before the commencement of the sickly season. Upon the same principle, sections of country which originally were very much infested with intermittent fevers, become healthy as a regular system of agriculture is introduced, and a considerable portion of the cleared land is converted into meadow and pasturage.*

4thly. The clearing of new land, in situations favorable to health is not necessarily followed by disease, and on every

* The climate of the northern and middle states becomes healthy as the country is settled, we presume from the causes we have just assigned. In the southern states, on the contrary, the system of agriculture appears to have a tendency to add to the disadvantages of the climate. Cotton and corn are the principal agricultural products of Georgia and the neighboring states; and these are planted for years in succession upon the same ground, almost without the intervention of any other crop. In consequence of this pernicious system of agriculture, the soil, deprived of the support of vegetable roots, and continually turned up by the plough, is carried by every rain into the neighboring low grounds to become, when exposed to heat and moisture, a fruitful source of disease.

large farm quantities of vegetable matter are left to decay in the vicinity of the dwelling without injury to the health of the inhabitants.

A long continuance of warm weather is necessary to give the exhalations of our marshes sufficient activity to produce disease. Hot and dry summers so far as my observations extend, are uniformly healthy. Marshes when perfectly dry are comparatively innocent. Heavy showers of rain during the summer, do not affect the health of the community, provided, they be followed for a few days, by cool, northerly winds. Those seasons are the most unfavorable to health which are distinguished by warm south winds, and a humid state of the atmosphere. If there be much rain under these circumstances the sickness is more general. Heavy rains during the sickly season put a temporary stop to the progress of disease; and the extent to which it may afterwards prevail, depends upon the subsequent state of the weather.

The appearance of frost does not, as is the case with the yellow fever, and is sometimes erroneously supposed to be the case with the bilious fever, entirely arrest the progress of the disease. The production of miasmata probably ceases at this time; so that strangers after this period are not liable to the fever, and probably, the system is so much invigorated by the change of weather, that with proper precaution there is less danger to those who have been exposed to miasmata.—But as the vicissitudes of the weather, and the change of temperature between the day and night are much greater; and very little care is generally taken to defend the system against these changes, the month of October is generally the most sickly in the year. The character of this fever is somewhat modified by this change in the weather, to which we shall refer hereafter.

The predisposition to intermittent fever remains, after exposure to its remote causes, for several months, even when the disease has not been excited into action. This is evident from the occasional cases of chill and fever we meet with during the winter, and from the remittent character of the inflammatory diseases of the winter. This predisposition probably ceases after a few months; vernal or summer intermittents verv rarely appearing except in persons who have suffered from the disease during the preceding fall. Such a case I have myself never seen: when they do occur, which from the testimony of other physicians, I have reason to believe is sometimes the case, the predisposition is probably kept up by the delicate health of the patient.

But when the disease has once been excited into action, the predisposition remains much longer. A severe attack of the fever generally leaves the system very much deranged, and until the health is restored the individual is more liable to a new attack, at an interval perhaps of one or two years. By successive attacks of this kind, the constitution is so much impaired, that a change of climate is indispensably necessary for the restoration of health.

This predisposition to intermittent fever frequently follows the individual into other climates; and when exposed to an exciting cause, he is liable to the disease in the most pure and elevated regions, after an interval of many months.

When the health is completely restored, and the functions of the animal economy are all restored to their healthy operation, the system is in some degree fortified against a new attack.

It is a favorite remark with physicians, in speaking of protracted intermittents, to say "they are kept up by the influence of habit." The propriety of this opinion, to the extent that is generally entertained, is questionable. In all cases of protracted intermittents the system is very much deranged, the pulse is quick, the appetite irregular, the secretious defective or vitiated, the complexion sallow, and the spleen enlarged. The writer of these remarks had a severe attack of bilious fever in 1826, followed by occasional attacks of chill and fever, until the next summer, and for several months after he was apparently restored to his usual health, the pulse continued from 10 to 20 strokes above the healthy standard. These facts show that there is something in the way of convalescence much more serious than a diseased habit. The tendency of the operations of the mind, and the actions of the body whether healthy of morbid, to form such associations with circumstances with which they are accidentally connected as to be re-excited by the recurrence of those circumstances, is well known; but such a cause can hardly be supposed to operate when the disease is renewed by exposure, after an interval of several weeks or months.

It is generally known to the profession, that trees or the brow of a hill form a very effectual obstacle to the diffusion of miasmata. Those planters who have adopted the precaution of leaving a quantity of uncleared ground about their houses, frequently enjoy an almost entire exemption from the attacks of this epidemic. It is not so generally known to the public, that elevated situations in the vicinity of marshes are not always an effectual security against disease. From want of attention to this subject, individuals, in selecting elevated sites for the sake of health, have been much disappointed, when by sheltering themselves under the brow of the hill their object would have been completely accomplished.

The situation of Milledgeville affords a very good illustration of these remarks. The town stands on the west bank of the Oconee, about a half mile from the river, upon two elevated ridges of land, separated by a small valley, and running nearly parallel with each other. The streets on the brow of the hill next the river are proverbially unhealthy, and frequently suffer severely from fever, while the other parts of the town are comparatively healthy.

These general facts are so familiar to the profession, that a very short account of the state of the weather during the last five years will be sufficient to illustrate the positions I have advanced.

The summer of 1824 was unusually dry. Between the 4th of June and the 20th of August, there was scarcely a shower of rain sufficient to lay the dust. During this period with the exception of some cases of inflammatory fever, and a few cases of bilious fever in low unhealthy situations, the country remained entirely healthy. At the expiration of this period, there fell a considerable quantity of rain, and the weather continued showery for several days. In a short time, sporadic cases of the fever began to make their appearance; many of them accompanied with dysentery.

The disease continued to spread until it received a temporary check from the appearance of the equinoctial rain in September. The country at this time was completely deluged with rain, and this again was followed by warm damp weather. Vegetation started forward with unusual rapidity, and the cotton before frost occurred grew to a size that is rarely seen in the most favorable seasons. In a short time the disease again began to spread; generally of the intermittent type, and frequently of an active inflammatory character, requiring very copious depletion. As the cold weather came on, many cases—among children whose clothing had not been accommodated to the changes of the weather, and among the laboring classes, who at this season of the year are alternately exposed, before sunrise to the dew of the luxuriant foliage of the cotton, and to the chilling air of the morning, and afterwardsthe sickening influence of an autumnal sun—assumed a low typhoid form which was frequently fatal.

The sickly season continued until the latter part of November when it gradually declined.

An observation made at this time,has been confirmed by the experience of each successive year, viz: that more children die of fever between the middle of October and the middle of November, than in any other month of the year; not excepting the ravages made by cholera infantum in June and July.

In 1825, May and June were excessively warm, although the heat of the weather was moderated by occasional showers of rain. During this period, the epidemic catarrh which had made its appearance in the northern states during the winter, and had been gradually making its way towards the south, appeared in Georgia, and prevailed very extensively. It was attended, in some cases, with high inflammatory symptoms, some of which, however, assumed the regular remittent type.

I expected that the symptoms of this epidemic would have been blended with the autumnal fever, but it disappeared in July before the commencement of the sickly season: and I did not observe that the few cases of fever which came under my observation received the slightest modification from this epidemic.

The remainder of the season was excessively dry, and as in the former year, the planter was compensated for the shortness of his crops, by an unusual exemption from disease. The equinoctial rains commenced on the 15th of September, and were followed as in the preceding year, by a good deal of sickness; generally mild intermittents, but a few cases were of a very malignant character.

In January, 1826, the epidemic catarrh again made its appearance, and spread very extensively, assuming every variety of that Proteus disease, and continuing to prevail during the spring.

The weather was exceedingly favorable to the agriculturist until about the first of July. After this, the occasional showers of rain were insufficient to keep vegetation in a flourishing state. Southerly winds prevailed in an unusual degree, and the humid state of the atmosphere was indicated by heavy fogs arising from the water courses. In June and July, inflammatory diseases, diarrhœa, and cholera, were very frequent. In the latter part of July, the bilious fever began to make its appearance, and by the middle of August, prevailed so extensively, as to invade almost every family exposed to the influence of its remote causes. Very heavy rains on the 10th, 11th, and 12th of September, followed by cool weather, and frequent showers, gave a temporary check to the disease, and very few new cases occurred, until within the last week in the month. At this time, it again began to extend; and so general was the sickness by the first of October, that it would be within the limits of a very moderate calculation, to say that two thirds of the inhabitants of extensive districts were confined at this time. In October and November, many cases appeared in the typhoid form.

It is remarkable that notwithstanding the general prevalence of the disease, very few cases terminated fatally until late in the fall. It is, I think, very frequently the case in healthy seasons, that the proportion of severe cases is very large. If this be a fact, does it arise from the greater concentration of miasmata in particular situations, or from the violence of the exciting causes, necessary to produce the disease?

The diseases of 1827 presented nothing remarkable, except the number of intermittents, and inflammatory diseases, which appeared in the spring and early part of the summer. The summer was dry, and remarkably healthy, except in some particular situations of small extent. Late in the fall, some severe cases of fever were occasionally met with.

The winter of 1827–28 was unusually mild, and the spring forward, and favorable to vegetation. On the 16th of April, when the forest was in full verdure, vegetation was destroyed by a frost so severe as to kill many trees of considerable size. From this circumstance, many predicted an unhealthy season. In May and June the weather was excessively hot, and the labouring classes, in consequence of the destruction of the crops by the frost, were exposed to unusually severe labour. From some one, or from the combination, of these causes, inflammatory fevers of a very obstinate character, were frequent; especially among the negroes; and in some instances terminated fatally.

During the summer, more rain fell, than in either of the preceding years; but it was uniformly remarked that every shower was followed for several days by cool northerly winds. The autumnal equinox was attended by high winds, without rain, and in consequence, there was an exemption from autumnal fever, that had scarcely existed within the recollection of the first settlers of the country.

After this account, imperfect, but I hope on a subject so familiar to the profession, sufficient for the object in view; I shall proceed with the history of the disease. So much has been written on the subject already, that I approach it with reluctance. It is however a subject of general complaint with the physicians of Georgia, that a work describing the peculiar character and treatment of this form of disease, is still a desideratum in the profession. Whether the disease assumes a different type, under different latitudes, or in different circumstances, I am not prepared to say; but I have certainly never seen a treatise on the subject which could qualify a stranger to treat it with success. It is my wish to confine myself entirely to its destructive peculiarities, avoiding all speculation, except what is necessary to establish that pathology, which I consider necessary to a correct treatment of the disease.

It is very important to make a distinction between those cases of inflammatory fever, which make their appearance early in the summer, and the remittent bilious fever arising from marsh miasmata. The former have no regular remissions, and are subject only to those exacerbations which usually accompany inflammatory diseases.

The latter is uniformly of the double tertian type, and the paroxysms of the fever recur with the greatest regularity; one paroxysm commencing in the morning, and manifesting a disposition to terminate in the evening of the same day; the other commencing on the afternoon of the following day and continuing through the greater part of the night, sometimes, until within a short time of the paroxysm of the next day.

There is a very marked difference in the character of these two paroxysms. That paroxysm which commences in the morning, is always preceded by a stage of depression, generally by a distinct chill; is attended by symptoms of greater congestion; with more determination to the stomach, brain, and other viscera; with more gastric irritability; and is far more violent and dangerous. The paroxysm which comes on in the afternoon of the next day, appears to consist more in simple nervous and febrile excitement. In mild cases, it is manifested simply by throbbing of the temporal arteries, restlessness, and anxiety. The memory and imagination of the patient are sometimes peculiarly active, revolving the past, and anticipating the future events of his life, with a degree of celerity very unusual to him; and it is very difficult to convince him that so active a state of the intellectual faculties is the precursor of a more violent paroxysm, on the succeeding day. In more violent cases, the fever is not so frequently preceded by a cold stage, is of a more open and inflammatory character than on the preceding day, and is attended with nervous excitement, rather than oppression or gastric irritation. It has a tendency to continue through the night, and seldom terminates by free perspiration. This paroxysm has a tendency to anticipate, and to become more violent, so, that if the case be protracted, it is sometimes difficult to distinguish 'the sick from the well day.' The distinction however may generally still be made, by the tendency of the one paroxysm to terminate in the evening, and of the other to continue through the night.

The first paroxysm may make its attack at any hour of the day or night; but, it is this paroxysm which, in the regular course of the disease, comes on in the morning. It is with this paroxysm, that the disease terminates in health, or the patient receives the shock which eventuates in death. Unless under very active treatment, the disease never terminates with the paroxysm which comes on in the afternoon; hence, if fever occur in the afternoon of 'the well day,' unless it can be traced to some accidental cause of excitement, or if the patient pass a restless night, we may confidently expect a return of the fever on the next day. The odd days may therefore be said to be critical.

The fever commonly arrives at its height by the 5th, 7th, or 9th day; and if the system is not prostrated in one of these paroxysms, we may *generally* predict a favorable issue. If the case is protracted beyond the eleventh day, the fever is in most cases kept up by some extraordinary local irritation, and a regular crisis seldom takes place.

The fever generally returns with a great deal of regularity. In violent cases it sometimes anticipates, and when it is disposed to a favorable issue, it sometimes comes on a little later. A little attention however, will enable us to calculate the hour of its return with sufficient accuracy.

It is of the utmost importance that the physician should be acquainted with this peculiarity in the course of the disease. While ignorant of this, the succession of the paroxysms appears involved in the most inextricable confusion; whereas, they are attended with so much regularity, that the judicious practitioner, with proper attention, can calculate with the utmost confidence, on the accession and termination of the paroxysm; can inform his patient at one time, that he will pass a restless night; and at another, assure him that with the assistance of an anodyne he will sleep comfortably.

I have lately been much gratified, in reading a work published by Dr. Jackson, in 1792, from observations made while surgeon in the British army in South Carolina, during the revolutionary war, to find that his remarks on the subject of the type of the fever coincide precisely with mine. These observations were made in a newly settled country, the soil and locality of which were very similar to the one I have just described.* It is indeed to me a matter of surprise that this peculiarity, which is certainly familiar to every physician conversant with the diseases of this section of country, should have been so seldom described.

I have stated that the paroxysm which commences in the morning, (and this paroxysm principally demands our attention, as the one in which consists the violence and danger of the disease,) is always preceded by a stage of depression, generally by a distinct chill. It is to this stage of depression that I wish particularly to direct the attention of my readers, since from it the fever derives its peculiar character, and its principal danger.

The bilious fever may be said to be a combination of intermittent fever, with inflammatory or irritative† fever. Such

---

* I omitted to mention that this territory was purchased of the Indians in 1805 or 1806, and consequently may still be called a newly settled country. Whether the bilious fever assumes the same type in other sections of the Southern states, I am unable to say.

† By inflammatory fever I understand those forms of general fever which are kept up by local inflammation. By irritative fever those in which it is kept up by local irritation, or an irritable state of the general system.

is the predisposition to intermittent fever at this season of the year, that every thing which tends to disturb the healthy actions of the system, proves an exciting cause. Consequently, the chill is, for the most part, the first in the train of morbid action; although when the disease is called into action by causes which tend to produce an inflammatory state of the system, it may not appear as a prominent symptom.

The stage of depression probably proceeds from a defective circulation in the capillary vessels; a retrocession of the circulating fluids from this class of vessels, and a consequent accumulation in the interior veins, laying the foundation for venous and visceral congestion. The chill, by the operation of some mysterious law, probably by an effort of nature analagous to the reaction which takes place after exposure to cold, produces the fever. The fever in its turn, after it has effected the object for which it was intended, viz: the restoration of the capillary circulation, has a disposition to terminate by perspiration, producing the sweating stage of the paroxysm.

Such is the regular course of intermittent fever; but this termination is liable to be prevented by a variety of circumstances, which prolong the paroxysm, and dispose the fever to assume the remittent form.

These causes are, 1st, The irritability of the system at large, arising from the influence of the climate, and, particularly, from the long continued heat of the weather; or of particular organs, arising from accidental causes conjoined with the effects of climate. 2d. The irritation from the congestions which take place during the chill. 3d. Local inflammation, either supervening upon the general fever, or arising from its usual exciting causes. 4th. And perhaps we may add to these, the derangements which take place in the capillary circulation during the chill. These are sometimes so great that perfect reaction does not take place, and it is probable under these circumstances, that nature interrupted in her remedial efforts, does not bring the fever to a perfect solution.

That the chill is the cause of the subsequent fever, we infer, from their invariable connection; from the fact, that when we keep off the chill we prevent the rise of the fever; that persons predisposed to chill and fever may, by exposing themselves to cold and thus producing an artificial chill at the usual period of its recurrence, bring on a return of the fever; and lastly, from the analogy of the effects produced by cold, where we find inflammatory action follow a state of the system very similar to the cold stage of fever.

The violence of the fever is not, however, strictly proportionate to the cold stage, because the fever is affected by a variety of accidental and constitutional causes, entirely unconnected with the chill. No one doubts that cold is the cause of inflammatory fever; yet who would pretend to predict the nature or violence of the fever, from a knowledge of the degree of cold to which the individual had been exposed.

That the fever proceeds from the chill, and not from local irritation, is evident from the fact, that in the greater number of those cases in which this irritation appears as a prominent symptom, it follows the fever and subsides in a short time after it. Thus, we observe the patient, who, during the paroxysm, has been tossing about the bed with all the distress attendant on gastro-enteritis, become calm as the fever goes off, and rest very comfortably until the accession of the next paroxysm. In those protracted cases of intermittent fever which are said to be kept up by the force of habit, the patient, immediately after the fever leaves him, eats his food with as good an appetite, and digests it with as little inconvenience, as the peasant who earns his bread by the sweat of his brow—facts that are certainly not easily reconcilable with much gastric irritation.

The stage of depression generally commences a considerable time before it manifests itself in the chill, and is indicated by a general defect of vigour in the system; languor; weakness; sensibility to cold; impaired sensation; sense of fatigue and aversion to exercise. At length it pervades the whole system and appears: On the surface, in the paleness, coldness and constriction of the skin, the impaired sensibility to irritants and impressions made upon the senses. In the muscles, by weakness, tremors, irregular action, and rigors.

In the stomach, by the nausea, sickness, vomiting, and insupportable anxiety.* In the internal surfaces generally, by a deficiency of their secretions. The increased quantity of bile, sometimes ejected from the stomach during the chill, forms an apparent exception to this general fact. It is to be remembered, however, that the liver is frequently the seat of great irritation from congestion and other causes, and thus is prevented from sympathising with the state of the circulation which prevails in other parts of the system. Further, the gall bladder is a reservoir in which the bile accumulates until it is required to assist in the process of digestion. This accumulation sometimes appears to be increased by a paralysis of the biliary ducts. For several days before the patient is confined, the bile is absorbed, probably from an obstruction of this nature, and carried into the system, tinging the skin and coats of the eye with its peculiar hue. The secretion of bile is very frequently diminished, and in violent chills sometimes completely suspended. A similar fact occurs in the cholera of the Indies; a disease which, in many respects, bears a very close analogy to the prostrate cases of autumnal fever. Lastly, this debility appears in the brain and nervous system, by the inactivity of the mind and the torpor of the senses.

The debility which attends this stage of fever is not the less real because it is not removed by stimulants. 1. Those stimulants do not indeed relieve it, which act primarily upon the heart; because they increase the action of the heart at the expense of the rest of the system, and thus increase those derangements of the circulation, under which the system is already oppressed. 2. Although the irritability of the system is diminished at the commencement of this stage, it increases when partial reaction begins to take place; and as those viscera will first receive the impression of stimuli, which are already too much excited, the general oppression may thus be increased. 3. Further, the debility is in a great

* To what cause are we to refer the thirst which accompanies the chill? It evidently proceeds from the stomach, and is accompanied with a peculiar sensation of internal heat.

measure owing to the interruption of the different functions of the animal economy, and, consequently, cannot be expected to yield to simple excitation of the vascular system.

Visceral congestion is the direct consequence of the recession of the blood from the capillary vessels. The spleen, the liver, and the brain, on account of the laxity of their texture, or the peculiarity of their circulation, are most liable to accumulations of this kind. The lungs are sometimes, but not so frequently affected. It is remarkable that when the chill is very distinct, the spleen is principally affected; but in those more dangerous chills, in which the sense of cold is a less prominent symptom, the engorgement takes place in more important viscera.

These local determinations deserve particular attention; for they hold so prominent a place in this form of autumnal fever, that they give rise to its distinctive symptoms. Every case of bilious fever may be said to be congestive, and requires a modification of the treatment adapted to this peculiarity. Congestion and inflammation are directly opposed to each other; the former bearing the same relation to the chill that the latter does to the fever. The balance of the circulation is disturbed in both. In the latter, there is a determination to the capillary vessels, increasing the sensibility of the system and the action of the heart; in the former accumulations take place in the veins, oppressing the functions of the viscera, and thus depressing the powers of the system. Under peculiar circumstances, however, either may imitate many of the symptoms of the other; and from a want of proper attention to this fact, inaccurate expressions, founded upon false analogies, have found their way into the writings of our most distinguished physicians.

As the history of the cold stage of intermittents is familiar to our readers, a few remarks illustrative of this peculiar form of fever will close our remarks upon this subject.

The chill is sometimes so violent as to prostrate the system by the mere debility accompanying it, without any particular evidences of congestion. Such cases are generally met with in old debilitated habits, or in persons who have been exposed to great fatigue, while suffering from the debility of a former attack. These cases are sometimes attended with very great danger, and require the utmost care to preserve the powers of life until reaction takes place. Two cases have fallen under my observation, in which the chill, after continuing between four and five hours and inducing the greatest debility, has terminated without reaction by a free perspiration. During the whole of this period, the vital organs were free from any oppression that would indicate serious congestion.

In the typhoid cases which occur late in the fall, the symptoms are very different. The chill is long, but without rigors or a very distinct sensation of cold, and accompanied with every indication of impaired capillary circulation. The pulse is small, irregular, and very quick; the skin is flaccid, generally, but not always cold; pale or sallow; the urine is pale and scanty; and the secretion of bile almost entirely suspended. The nervous system is equally affected. The external senses are impaired; the mind is sometimes bewildered and confused, like that of a person suddenly roused from profound sleep; more frequently, the patient sinks into a state of stupor from which he cannot be roused. The reaction is here very imperfect; the pulse does not increase in fulness when the fever rises; nor does the skin recover its elasticity; and the extremities remain cold during the whole paroxysm. These cases are frequently and most unexpectedly fatal. The disease is so insidious and the patient so little disposed to complain, that the friends are not aware of his danger until he is irrecoverably prostrated. The chill is not always very distinctly marked in cases of this kind; it can never, however, escape the observation of the most inattentive practitioner.

Congestion frequently takes place before the cold stage is observed, and in plethoric or irritable habits, produces so much local irritation, that the stage of depression may even pass unnoticed. In common language, the fever is here said to rise before the chill. Though the symptoms of this stage are obscure under such circumstances, they may generally be recognized in the languor, oppression of the brain, the weakness and irregularity of the pulse; the coldness of the extremities; and the irritability of the stomach; very often too, where this stage has passed unnoticed in preceding paroxysms, the patient is suddenly prostrated at the very commencement of the paroxysm. The chill is generally accompanied by pain in the head, back and loins. The pain in the head sometimes appears to be a mere nervous affection, that is promptly relieved by the administration of a diffusible stimulant, or the application of a sinapism. More frequently, however, it arises from congestion, and continues throughout the paroxysm. The pain in the back and extremities is generally most violent in those cases which are attended with severe agues; and which are seldom dangerous. One fatal case, however, has occurred to me, in which the patient complained principally of excruciating pain in the ancles.

Occasionally I have seen the chill accompanied with great depression of spirits which subsided as the fever rose. In one case it returned on the 3d and 5th day, and continued through the usual period of the chill, (although this was prevented,) subsiding as before on the appearance of the fever. An intelligent friend, to whom I mentioned this circumstance, informed me that he had observed a similar phenomenon in the depression which follows severe wounds; the mental depression here also subsiding as inflammation supervenes. In the cases to which I allude it was attended with great hepatic congestion; a circumstance which may have had some influence upon the state of the mind.

Cases of bilious fever have been described as analogous to the congestive forms of typhus and fellow fever. If such cases do occur, I am disposed to think the congestion is subordinate to the chill, and is accompanied with so much real debility, that the bold practice of depleting, sometimes resorted to in the former diseases, for the purpose of relieving oppression, is totally inadmissible.

The history of the symptoms, we think, will justify the view we have taken of the chill, and the importance we have attached to it. The chills may be long and violent without much danger, because the derangement of the circulation is principally confined to the surface. The capillary vessels of the stomach may be principally affected, and sickness and vomiting will be the prominent symptoms. The symptoms of congestion are sometimes more conspicuous than those of the chill; and the stage of depression is marked by stupor, difficult respiration, &c.; and in some instances, so much local irritation is produced that the symptoms of this stage are very obscure.

In other cases the whole system is simultaneously attacked, and the patient prostrated.

We have assigned four causes, which tend to protract the paroxysm, and dispose the fever to assume the remittent form.

1. The determinations to the viscera which take place during the chill, prove a source of irritation and tend to protract the paroxysm. Congestion, although in its nature the opposite of inflammation, generally produces local irritation. The symptoms of these local determinations are very apparent in every violent case of bilious fever, and may be said to impart to it its peculiar character. To these we are to attribute the pain in the head, stupor and convulsions; oppression of the chest; enlargement of the liver and spleen, and unequal distribution of heat and excitement. Hence the pulse at the wrist remains unaltered during the successive stages of the paroxysm, while the action of the carotids, and other symptoms give evidence of the most violent internal excitement. When these symptoms appear the remission is seldom distinct; the fever seldom terminates by perspiration, and tonics can rarely be administered with propriety.

2. The second cause to which we have referred, is the irritability of the system, which prevails at this season of the year, from the influence of long continued heat. From the influence of this cause, bilious fever is characterized by great irritation, rather than inflammatory action; and this peculiarity increases, for the most part, as the season advances.—Hence in violent cases, the pulse is always very frequent, and late in the season bleeding must be used with more caution, and exerts less control over the disease, than in other cases of inflammatory fever.

The influence of hot climates, in deranging both the functions and structure of the liver, is familiar to the profession. Accordingly, we find in almost every case of this fever, that the secretions of this organ are vitiated, indicating that it has become a centre of irritation, to which the physician should particularly direct his remedies, and against which he should always be on his guard.

The stomach, if we may judge from the capricious state of the appetite in warm weather, is not less affected by the climate than the liver. When this obvious indication of nature is counteracted by condiments and stimulating food, the sensibility of this organ is so much increased, that it is kept in a state of constant irritation. Hence the affections of the stomach and bowels so prevalent in the early summer months, and which continue until the system is habituated to the state of the weather, or new causes of disease have arisen to divert morbid action into other channels. Under these circumstances therefore, when we consider the sensibility of the stomach, and the irritation to which it is continually exposed from excessive and improper diet, it is not a matter of surprise that it should suffer very much in this disease. A peculiar affection of its mucous coat marks one of the most violent forms of the fever. It is tender upon pressure, excessively irritable, with great thirst, and that feeling of distress and anxiety usually present when its functions are deranged. The pulse is small and frequent, the skin pale, and frequently delirium also is present. Since this affection subsides with the fever, so far that bark may very frequently be freely given in the intermission, it is, probably, merely the result of general inflammatory action, upon an irritated and exquistely sensible surface.*

* An affection of the stomach of a very opposite character is sometimes produced by the administration of calomel or tarter emetic; especially if administered during the chill. The distress, restlessness and anxiety are the same, but the prostration of strength is much greater, and the disposition to vomit incessant. It is less dangerous however, for if not relieved by warm water. it is readily controled by an anodyne. In a succeeding paroxysm this irritation, the effect of medicine or of the chill, frequently is followed by the inflammatory irritation described above.

3. Those organs which are the seat of the congestion, or of great irritation, in protracted cases, are frequently affected with local inflammation, especially if they are either constitutionally weak, or suffering from the effects of previous disease. The lungs, in those who have had repeated attacks of pleurisy; the stomach, in those whose constitutions have been injured by free living, or who have suffered from that species of fever which prevails on the seaboard; the liver and spleen, in those who have had intermittent fever in marshy districts.

In delicate females, who have passed the meridian of life, and whose constitution has been impaired by repeated attacks of fever, we frequently meet with an erysipelatous inflammation of the stomach. In addition to the symptoms usually attendant upon affections of the stomach, the whole alimentary canal is frequently affected by continuous sympathy; and the tongue is either covered with a brown fur, or is hard and dry as if it had been baked; or the whole surface of the mouth is of a florid colour, and very tender. When the bowels are affected we have serous diarrhea. These symptoms generally indicate a protracted case, the more troublesome, because it admits of no active treatment.—These unpleasant symptoms are sometimes produced by the injudicious administration of tonics.

The predisposition to bilious fever is so strong that all other diseases prove exciting causes. It may thus be combined with a variety of inflammatory affections, dysentery, puerperal fever, &c. When the causes of inflammation act very powerfully, the remissions of the fever are very obscure, until the inflammation is subdued. We have here, I think, a very evident departure from the adage; 'that epidemics compel all other diseases to obey their laws.'

4. We have assigned imperfect reaction as an additional reason for the protraction of the paroxysm. Upon this point we have some hesitation. In most of the cases, in which a cause of this kind might be supposed to operate, the fever may possibly be kept up by congestion. In those cases of intermittent fever which proceed from long continued exposure to cold and moisture, as in the case of labourers who are exposed to the morning dew in picking cotton, the intermission is very distinct, and there is apparently no reason to suspect any latent cause of irritation; yet the fever terminates without perspiration, and tonics are productive of little advantage. Does there not appear under these circumstances to be an interruption in the regular process of nature, which prevents her from effecting a perfect solution of the paroxysm.

These are the causes so as we are able to trace them which tend to protract the paroxysms.

Accordingly, we may expect to find that the fever will assume more or less of the remittent character, according to the circumstances of the patient, or the nature of the exciting causes. Thus, if the disease has been excited by exposure to the cold evening air, we may generally expect the intermittent form of the disease. If exposure to the rays of the sun, or errors in diet, have been the exciting cause, we may expect the fever to approach the continued form. In individuals of plethoric habits, the first three or four paroxysms run into each other so much, that the remissions are scarcely perceptible; and under proper treatment, or simply from the tendency which the fever has to wear itself out, may terminate in an intermittent form. Other cases which are strictly intermittent at the commencement, from neglect, or improper treatment, become complicated with so much local irritation, that they approach very nearly to the continued form. If the first attacks have been properly and efficiently treated, I think, as a general rule, relapses are more apt to assume the intermittent form. In children, the remissions are less distinct than in adults, and if the fever is accompanied with convulsions, the regular type of the disease is so much interrupted, that the return of the paroxysms cannot be calculated with any accuracy.

The pulse, as might be supposed from the Proteus form of the disease, varies much. It is sometimes full, active, and strong; more generally, from the congestive character of the fever, it is small and weak, affording no satisfactory indication of the extent to which depletion ought to be carried. It is frequently the same in every stage of the paroxysm; the small weak pulse which distinguishes the chill, continuing through the hot stage, and perhaps becoming more full and free, as the fever subsides. Under other circumstances, I have seen the pulse, during the chill, so active as apparently to justify copious bleeding, and continuing without any increase of strength, when the action of the carotids became so violent, that their pulsations were visible across the room. In violent cases, from the great irritability universally attendant upon them, the pulse is always very quick.

The state of the secretious is various. The tongue, at the commencement, is generally covered with what appears to be a secretion of white mucus; which is altered as the disease advances, according to any accidental turn it may take. During the chill, all the secretions are suspended, except the bile, which is frequently thrown up in great quantities. In some cases of great prostration this secretion is suspended also, and in others even of an active inflammatory nature, it appears to be defective; the most active cathartics producing no bilious evacuations for several days.

Delirium is a frequent attendant upon bilious fever, generally, however, accompanied with a disposition to coma. Although congestion for the most part has a tendency to produce coma, rather than furious delirium, yet I have seen a few cases in which the chill was ushered in with the wildest form of mania. A similar fact sometimes occurs as a consequence of congestion under other circumstances. Delirium generally attends great irritation of the stomach; and is distinguished by the distress, restlessness, and anxiety, which attended it.

Convulsions frequently accompany the disease in children. They generally appear in the hot stage, or just at that period when the chill is terminating in the fever; and in a few instances, I have met with them in the commencement of the chill. They are generally to be considered as a very alarming symptom. In a very few cases they accompany the fever in adults.

A severe attack of fever always leaves the system very much deranged. All the secretions are impaired; the skin is dry and harsh; the biliary secretion alternately vitiated and defective; the bowels constipated. If the case have been protracted, there is always a tendency to anasarcous effusions; and, if the chylopoietic viscera are enlarged, very frequently to ascites. The liver and spleen are often liable to a kind of slow inflammation which frequently accompanies the patient for many months. Irregular fever returns on the slightest excess and exposure. Sometimes the health is so much deranged that it can only be restored by a change of climate. All these evils are aggravated by an ungovernable appetite, which the considerations of prudence, the remonstrances of friends, and the authority of the physician, are equally ineffectual in restraining.

TREATMENT.

*Bleeding.*—The propriety of resorting to this remedy, and the extent to which it may be carried, depend very much upon the season of the year. Early in the season, when the pulse is frequently active and strong, it may be repeatedly called for, and resorted to with great freedom. As the season advances, and the fever assumes more of the character of irritation, greater caution is necessary; indeed the fever is beyond the power of the lancet, and the patient would sink under the loss of blood, before we reach the seat of the disease.—This evacuation is often highly necessary, when the pulse gives a contrary indication. Indeed, the pulse seldom affords an accurate criterion of the extent to which depletion is demanded; and we examine it, rather to determine whether the system will bear depletion, than with the expectation of obtaining any precise information, as to the extent to which it ought to be carried. On account of the oppression of organs essential to life, it is sometimes necessary to bleed to an extent that the system, in this deranged state of the circulation, is little able to bear; and accordingly, efficient means for equalizing the circulation such as sinapisms, and the warm bath, and sometimes diffusible stimulants, should be resorted to at the same time. Under these circumstances, the abstraction of 10 or 15 oz. of blood, by means of cups or leeches, will sometimes produce the happiest effect. In congestive cases, I have never ventured upon bleeding, until general reaction had taken place. When the stage of debility is so violent, that the system is prostrated, and the surface remains cold, the idea of depletion cannot be entertained for a moment.

On account of the short duration of the paroxysm, perhaps we are justifiable in carrying depletion to a greater extent, than we would be willing to do under other circumstances. The paroxysms of fever resemble a hurricane, violent, but of short duration, through which if the patient can be conducted in safety, he has time to recruit his energies before the next attack.

*Emetics.*—These are peculiarly useful in bilious fever. As an evacuant of the morbid secretions of the liver, they are inferior only to calomel; and this effect they have, whether they excite full vomiting, or only nauseate. They relax the surface; promote the secretions generally; shorten the paroxysms; and frequently break up the disease. As they are occasionally, however, attended with unpleasant effects, some caution is necessary in directing their administration.

1. If high febrile action or much determination to the head exists, they should be preceded by other modes of depletion, or be given in divided doses at long intervals, that by the relaxation they produce, the increased determination to the head may be avoided.

2. The practice, recommended by some authors of giving them, either immediately before, or during the cold stage, with a view to prevent or shorten it, has, in the hands of ignorant practitioners, been attended with the most disastrous consequences. That reaction, and equalization of the circulation, which these gentlemen expect, never take place; and they produce great debility, obstinate vomiting, and spasms of the stomach.

3. They should be given with caution, when the stomach is very irritable. This irritability arises less from any irritating substance that an emetic might remove, than from the effects of the chill. It is generally increased by emetics, and the most we can do with propriety is, to promote vomiting, by warm water, or infusions of eupatorium, or chamomile.

If this disposition to vomit be an effort of the *vis medica trix naturæ*, it is one which we would rather discourage than promote. For this purpose, a variety of arom atics; carbonate of ammonia; opium; lime water, and external irritants, are used. Sipping water, as hot as it can be borne in the mouth, frequently, has a very good effect.

4. Emetics should not be given, when from idiocyncracy, habit of living, or the debilitated state of the health, we have reason to believe they will operate unkindly. When it is necessary to give them under these circumstances, a full dose of some mild emetic should be given at once; or the system should be prepared for their operation, by an anodyne taken some time previously.

5. They are injurious in all cases in which the reaction is incomplete. In those typhoid cases which we meet with late in the fall, I have never known a child recover to whom one had been administered.

Emetics are frequently given in nauseating doses, in such a manner, as to make a very decided impression upon the stomach. Whatever may be the propriety of this practice in other febrile diseases, it is recommended in the bilious fever by the following considerations: 1. Emetics are useful in this disease by their specific operation; and here, it is probable that too much has been attributed to their mechanical effects. Nauseating doses of emetics, especially if their purgative operation is promoted, sometimes evacuate the secretions of the liver, and even relieve jaundice as effectually as if they had operated by vomiting. 2. The paroxysms of the fever are short, and disposed to terminate by free perspiration; by a liberal use of this class of medicines, we hasten this event, without the inconvenience of subjecting the patient for a length of time, to this unpleasant mode of treatment. 3. Whatever may be the rationale of the practice, it is confirmed by the universal experience of the southern physicians; who are in the habit of applying the same mode of treatment, with the most decided advantage, to the inflammatory diseases of the winter.

*Cathartics*, it is unnecessary to say, are indispensable in almost every case of bilious fever. We occasionally meet with cases, attended with so much prostration, that we are, for a time, prevented from resorting to them; but we can seldom dispense with free evacuations in every paroxysm, until the secretions from the intestines resume a healthy appearance. As a purgative, calomel, from its mild and effectual operation, and its influence over the secreting surfaces, is superior to any other cathartic. It should be given in doses of from ten to twenty grains, and, after an interval of five or six hours, followed by some active cathartic; and for this purpose, a combination of the more stimulating cathartics is generally preferred. After the bowels have been freely evacuated, the time of the paroxysm should be selected for evacuating medicines of every kind. When given at this time, they produce less debility; moderate the fever; and afford the patient, during the intermission, time to recover from the effects of the preceding paroxysm. When we wish the calomel to make a strong impression upon the system, it may be given in the morning before the paroxysm; and after the fever rises, the purgative above mentioned should be given in divided doses, so that they may operate effectually, with as little irritation as possible. This precaution is very necessary. When given in large doses, purgatives, although they produce copious discharges, create so much irritation, that the intestines are imperfectly evacuated. Hence the most active cathartics frequently have but a temporary effect in reducing fever, until by the administration of calomel, we succeed in discharging those vitiated secretions which appear to have escaped the operation of other medicine. Hence too, the saline cathartics, however actively they may operate, are the most inefficient of the whole. So much have I been impressed with the importance of this observation, that when I have observed calomel produce copious serous evacuations, I have frequently checked its operation, and repeated the dose with opium. We sometimes derive great advantage from giving opium with calomel. When the extremities are cold, with other indications of imperfect reaction; when the system has been improperly reduced by bleeding, or other evacuations; and lastly, when we wish to obtain the peculiar effects of this cathartic in persons, upon whom from some idiosyncracy it operates unkindly, without subjecting the patient to the inconvenience which might result from its use.

We occasionally meet with persons who have taken so much calomel, that it no longer produces its specific effects, but runs quickly through the bowels, producing, like the saline cathartics, copious watery discharges. Upon some delicate persons, it produces the most distressing symptoms of gastric irritation; restlessness; anxiety; insupportable sense of sinking, even fainting. These effects are independent of the evacuations produced; frequently occurring in a short time after it has been received into the stomach. By allaying the excessive irritability of this organ, we give the calomel time to operate; and by means of other cathartics, can carry it off at our leisure.

If calomel is given during the paroxysm, from two to five grains combined with a nitrous powder, may be given, at proper intervals, until a sufficient quantity has been taken, to be followed by some other cathartic. This combination is a very powerful one; and certainly has an admirable effect in moderating the violence of reaction, and bringing the fever to a solution. In delicate constitutions, it requires to be used with some degree of caution, on account of the violence with which it occasionally operates.

The calomel is to be continued, according to the violence of the disease and the tendency to visceral inflammation, taking care, if possible, except in particular cases to which we shall presently allude, to avoid salivation, which is rarely necessary, always distressing both to the patient and physician, and frequently a serious obstacle to a rapid convalescence.

In cases attended with great congestion, when much depletion would be improper, it is freqently advisable to repeat the doses of calomel until they operate, without the intervention of other purgatives. It will rarely, under these circumstances, produce salivation.

After the combination of active cathartics to which I have alluded above, I have next preferred castor oil; which combines the property of operating effectually where we wish an active catharthic, and so mildly, as to be suited to the most delicate constitutions. With the operation of the saline medicines, I have been so much dissatisfied, that I have almost entirely proscribed them. Seidlitz powders, however, form a useful assistant to other medicine; and in mild cases, where the principal indication appeared to be to restore the secretions of the skin, I have given epsom salts with an infusion of snake root, perhaps with advantage.

*Salivation* may occasionally be resorted to with advantage. When the liver and spleen are inflamed; when the pain in the head is very obstinate, continuing during the remissions, so as to lead to the conclusion that the membranes of the brain are inflamed; and perhaps where the stomach and intestines are affected with great irritation; if these symptoms do not yield to the antiphlogistic treatment, combined with mercurial purges, it will be advisable to continue the administration of calomel until the mouth is affected.

There is said in inflammatory diseases to be a blistering point. The salivating point is still lower in the scale of action. Until we reach this point, calomel should only be given as a purgative; and after this, it will still be advisable to give it in full doses, combined, if necessary, with opiates, and repeated according to the urgency of the symptoms. Experience has proved that a salivation, brought about by full doses of calomel, exerts a much more decided influence over inflammatory affections, than when produced by smaller doses. The difference in its effects is almost as conspicuous as in bleeding from a large orifice in a sufficient quantity at once, or taking the same amount of blood at different periods.

Dr. Cartwright supposes that salivation, when produced by full doses of calomel, is not so apt to proceed to a distressing extent, as when produced by smaller doses. The same remark, I think, will apply to the combination of calomel with opium. I have given, in one instance, 20 grains of calomel with 2 grains of opium, every second night, for three weeks, without affecting the mouth. On other occasions, I have given the same quantity of calomel with 1-2 grain of opium, at intervals of five or six hours, for two or three days, with a very slight affection of the mouth.

With respect to *cold water*, it is customary when the fever is high, to allow the patient to drink and apply it to the surface of the body *ad libitum;* except, perhaps, in a few cases, where from the delicacy of the patient, we have reason to apprehend unpleasant effects after the administration of some kinds of medicine. I have occasionally met with patients who affirmed, that cold was less efficacious in relieving pain in the head, than warm fomentations. In some cases in which the headache proceeds from congestion, this is probably the case; and if cold applications are continued after perspiration begins to appear, they certainly prolong the headache.

*Blisters* are not unfrequently a very important remedy. When the local affections depend upon congestion, and proceed from the effects of the chill, they are frequently so urgent as to require the most prompt methods of relief; and under these circumstances, sinapisms of a large size, are more speedy, and probably more efficacious, in their operation. But where the congestion has become fixed, and is combined with some degree of inflammatory action, as it generally is in such cases, they are too transient and superficial in their effects to be relied upon; and blisters are therefore to be applied, as soon as the reduced circulation and the state of the paroxysm, will justify. Very frequently, the sensibility of the surface is so much impaired by the oppression of the interior organs, that blisters may be applied during the paroxysm, at a very early stage of the disease.

When intermittent fever is accompanied with much irritation in the abdominal viscera, by applying the blister so as to obtain its full effect, at the period of the recurrence of the chill, we sometimes secure the double effect, of preventing the chill, and relieving the visceral affection. This time is not, however, on the whole, favorable for the application of blisters. Circumstances seldom permit us to wait for this period; and if the blister is suffered to remain until the fever rises, it proves very distressing.

When the head is affected, they may frequently be applied with advantage, to the stomach or extremities. The stomach is always very seriously affected, and is more frequently the centre of diseased action than is generally suspected; and consequently, we will often find morbid action in distant parts of the system, promptly relieved, by remedies directed to this organ. In cases of great congestion, it is frequently important to multiply points of irritation, by applying blisters both to the stomach and extremities.

Since there is so intimate a connection between the chill and fever, it is always a matter of great importance to prevent the former. For this purpose, our most effectual remedy is *opium*, in its different modes of preparation. Many objections have been made to this remedy, from the injudicious manner in which it is frequently used; but by attention to the following rules, it will be found a most valuable remedial agent, which, in many cases, is indispensable.

1. It should not be given until the system is prepared for its use, by purgatives or emetics. There is so close a connection between the state of the viscera, and the surface, that medicine will seldom act favorably upon the latter, until the secretions of the former are restored, and all causes of irritation removed from their mucous surfaces. There are some exceptions to this rule, to which we shall presently refer.

2. It should not be given until the inflammatory disposition is subdued by bleeding, or other evacuants. When the cold stage is not distinctly marked, or so much irritation exists, that the fever rises before the chill, it should rarely be given. Some authors consider it equally useful, whether the fever is preceded by a cold stage or not. I have only to add, that I have never employed the remedy under these circumstances, except in protracted cases, in which the intermission was very distinct, and the fever, without any local affection, appeared to be kept up by general irritability. In such cases, Dovers' powder will frequently prevent the return of fever

3. It should be given in a full dose,* and sufficiently early, to bring the system completely under its influence before the time of the expected paroxysm. There is more danger from an inefficient dose than from a large one; because the former, while it does not prevent the fever, acts as a local irritant, without the benefit of that relaxation, and determination to the surface, which proceed from a full anodyne. When delayed until the chill makes its appearance, it had generally better be dispensed with; and other means adopted to carry the patient through the chill, as stimulants, under these circumstances, increase the subsequent fever, without relieving the chill. An exception may perhaps may be made of those cases, in which the system is prostrated under the chill. Very powerful stimulants are sometimes necessary, when the irritability of some parts of the system is so much increased, that they can hardly be administered with safety. A large anodyne may here be necessary, to enable us to reap the full benefit of more diffusible stimuli.

4. It will frequently be advantageous to combine the opium with small doses of ipecacuana or tart. antimo., when the stomach is not already too irritable. Dovers' powder is with many a favorite prescription; but as the mere taste of this medicine frequently causes its immediate rejection, I have generally preferred the combination of tart. antimo. with laudanum, as less apt to offend the stomach, and from its liquid form, more prompt in its operation.

5. The beneficial effects of the anodyne should be secured, by the administration of aromatic infusions, or diffusible stimulants. When the debility was very great, and it was deemed particularly important to prevent the chill, I have endeavored to anticipate the cold stage the more completely, by giving carbonate of ammonia or wine whey, sometime before the opiate.

If opium should fail to prevent the chill, it does not, when judiciously administered, have the effect of increasing the

* Some practitioners prefer giving it in divided portions, so as to keep the system under its influence until the period for the chill is past.

fever so much, as from theory we might be led to expect. It prevents congestion or renders the system insensible to its irritation, and disposes the fever to terminate more speedily, by a free perspiration. An unfounded prejudice against this medicine has arisen, from its producing such an unpleasant confusion of ideas during the fever, that although the paroxysm is shortened, it appears to the patient interminable.

The infusion of *capsicum* has lately acquired a very high reputation, from its efficacy in preventing the chill. A strong infusion is given in doses of a wineglass full every hour. It very promptly removes the sickness attendant upon the chill, and is probably superior to the carb. of ammonia under similar circumstances; but its beneficial effects have been much overrated. Upon this point, I can speak with the confidence of personal experience; having taken it freely under circumstances the most favorable for its administration, with no other beneficial effect, than that of relieving nausea; and if the statement of other physicians is to be credited, its indiscriminate use has been productive of a great deal of mischief. As it increases the force of circulation very little, it probably exerts an influence through the medium of the nerves of the stomach, by an operation very analogous to that of rubefacients applied to the skin. Its taste is not so acrid as we should expect; and the impression left upon the fauces is immediately removed by chewing a little bread.

*Sinapisms* are a very important means of preventing the chill, and relieving the unpleasant effects which accompany it. They should be applied of very large size, to the extremities and over the whole region of the stomach, about an hour before the return of the paroxysm, and suffered to remain until they make a decided impression. Where reaction is very imperfect, as in the typhoid cases which we meet with in the fall, and among the miserable inhabitants of low marshy districts, they are very useful, and sometimes form our principal resource. In removing the sickness, oppression, and stupor, in these cases, they are truly invaluable. Dr. Daniel, of Savannah, has lately attempted, (and by the way with too much success,) to introduce pepper and mustard into general practice, to the exclusion of almost every other remedy. I have not leisure to point out the absurdity of his theoretical reasoning; nor to shew, that from the cases he has advanced in defence of his practice, he has no right to extend it farther than the limits I have laid down.

I shall merely take occasion to remark, that although the peculiar nature of the bilious fever in the southern states, requires irritants, (*not blisters,*) to be applied to the skin more freely than in other febrile diseases; yet the fact that external irritants of any kind, during a state of high excitement, will certainly increase febrile irritation, is too well established to admit of argument. And again, the importance—the absolute necessity of calomel as a purgative in the diseases of southern climates, is so fully acknowledged by intelligent physicians of every variety of character, by the cautious practitioner as well as the bold innovator, that it requires the experience of more than one individual, to produce even a doubt on the subject. There are cases in which calomel cannot be used at the *commencement* of the disease; but a judicious use of this medicine will always expedite convalesence. If it were only to obviate the tendency to visceral disease, it would still be invaluable.

Upon what principle is it, that external irritants fulfil so many important, and apparently opposite indications; that they reduce inflammation; remove congestion; restore the balance of the circulation; and in the stage of collapse support the sinking powers of life? Is it not by a uniform mode of operation, viz: by imparting tone and increased action to the capillary vessels? If applied too early in inflammatory affections, they are uniformly injurious; but when arterial action is reduced, they enable the capillaries to throw off the load by which they are oppressed. In congestion, by the same mode of operation, they increase the action of the heart, and remove the obstacles to the circulation of the blood; and when the system is worn down by great excitement, they afford a new stimulus to sustain the powers of life, without unnecessarily exciting an organ, whose excessive action has already exhausted the system.

When a distinct intermission of a few hours takes place, after the stomach has been cleansed, we resort to tonics; and the *Sulp. of Quina* appears to combine the advantages of all these, with a freedom from many of the objections, to which the most of them are liable. It is easily taken, is quickly dissolved in the fluids of the stomach; and if it does not prevent the return of fever, will not remain, as is frequently the case with the bark, a permanent source of irritation during the hot stage. Some intelligent practitioners of great experience, have informed me, that they generally gave it without hesitation, whenever they discovered a distinct intermission, without discovering any inconvenience from its liberal use. I am not prepared to go so far; for I have certainly seen very serious inconvenience follow its administration, even when there was a perfect intermission of twelve hours, with a soft pulse, and a general relaxation of the surface. Some caution is undoubtedly necessary, therefore, in resorting to it.

If there be much oppression about the precordia, or any visceral irritation or inflammation, whatever, remaining during the intermission, it had better be avoided. If recent enlargements of the spleen exist, or if long standing affections of this kind have increased in size and induration, they will generally be found to keep up so much irritation, as to render it ineffectual: and when the fever does not terminate by perspiration, we may generally suspect some irritation of this kind to exist. Under these circumstances therefore, it will generally be more prudent to resort to other means to prevent the return of the paroxysm; or if we try it in urgent cases ineffectually, to discontinue its use. Opiates are, under these circumstances, generally safer and more effectual.

We sometimes meet with cases, of a mild nature, in which, during the intermission, the skin is dry and harsh, and the pulse small, and feeble, that are best treated by diaphoretics, warm infusions of eupatorium or chamomile, assisted by Dovers Powder, or carbonate of ammonia, and the warm bath. This diaphoretic mode of treatment would probably be the most effectual we could pursue, 'in the cold cases,' if we were called sufficiently early to anticipate the paroxysm.

By the subsidence of the paroxysm, the system is frequently left in a state of great debility. A full anodyne, to allay the excessive irritability of the system, is frequently attended with the happiest effects. It is not generally prudent to give it, until the fever subsides; but sometimes it will bring a protracted paroxysm to a termination. The liberal administration of wine whey, carbonate of ammonia, and even of the more powerful stimulus of alcohol, is frequently necessary to sustain the system, in the great collapse which sometimes takes place.

During convalescence, much prudence is necessary to reestablish the health. The state of the bowels demands constant attention. If there be not much fever present, warm stimulating purgatives as the tinct. myrrh, and aloes, &c. are very useful. But if these prove too stimulating, castor oil, or rhubarb, may be substituted. If any particular local irritation remain, alterative doses of calomel may be given, or occasional purgative doses of calomel may prove necessary.

Tonics generally do not promote convalescence much.—When resorted to, it should be in combination with some vegetable diaphoretic. The infusions of virginia or seneca snake root, taken in large quantities, are frequently used in domestic practice, for the purpose of removing anasarcous swellings; and under some circumstances with a very happy effect. Protracted intermittents are frequently broken up by emetics, a mode of evacuation which is recommended, by its invigorating, instead of debilitating the patient. A succession of mild emetics taken every second or third day, for some days, will frequently be very useful. Some cases accompanied by continual fever, so mild as not to keep the patient confined to the bed, I have relieved by a solution of tart. of antimo. in doses sufficient to operate upon the bowels, without producing nausea.

In the derangement of the general health which follows protracted cases, some physicians place great confidence in alkaline remedies, especially lime water. Hence it is customary to recommend patients to visit Tennessee, for the benefit of the lime stone springs, which abound in that state. Whether it is from this, or from travelling in an elevated mountainous country, that so much benefit is derived, I am not prepared to say. From the good effects of this class of medicines in other dyspeptic diseases, I think it probable that the lime water deserves a portion of the credit.

Chronic enlargements of the spleen are often very troublesome. They are always connected with a bad state of health, and always disappear as the general health is restored. The rapidity with which they are dispersed is sometimes almost incredible. When accompanied with local irritation they should be treated with mild laxatives, rhubarb and aloes, and occasional purgatives of calomel; after this is subdued, alterative doses of mercury, tonics, gentle emetics, vegetable alteratives, and exercise as active as the strength will admit of. Dr. Drake's prescription of tonics with diuretics is a very happy one. This last class of medicines has a general effect upon the secreting surfaces, and this is precisely the kind of operation we wish. When inflammation has subsided, salivation is perfectly useless for the purpose of reducing the enlargement.

When other remedies have failed, or the patient is unwilling to resort to them, protracted intermittents are generally relieved by Fowler's solution. It is now, I believe, well understood to be applicable to protracted cases only, and to require during its administration as strict an attention to the state of the circulation as if the patient were put upon the use of bark. Without this precaution it will frequently fail. In a physician of my acquaintance who incautiously took 25 drops at a dose, it produced a slight salivation. Whether this was an affection of the gums similar to the effect produced by mercury, or an affection of the mucous membrane of the mouth, from sympathy with the impression made upon the stomach, I am not, at this distance of time, able to say.

In cases of dropsical effusion, when other remedies have failed, I have prescribed the tinct. of digitalis with uniform success. As the patients for whom I have found it necessary to prescribe it, have been of intemperate habits, or debilitated by disease and the operation of other remedies, I have commonly combined it with opiates.

The morbid appetite which follows bilious fever, I have been led to suppose, arises, frequently from such a depraved condition of the sensibility of the stomach, that it does not afford a criterion of the quantity of food that is necessary. Hence it is rather diminished, than increased by tonics. At the same time, the mind of the patient should be diverted by some light and agreeable occupation.

In conclusion, it may be remarked that the state of the bowels, during the convalescence, cannot be too carefully attended to; and that, as a general rule, emetics, in the sequelæ of bilious fever, display the best effects that, under any circumstances, we are to expect from them.

*August*, 1829.

